# Genetic Diversity of *Plasmodium falciparum* in Haiti: Insights from Microsatellite Markers

**DOI:** 10.1371/journal.pone.0140416

**Published:** 2015-10-13

**Authors:** Tamar E. Carter, Halley Malloy, Alexandre Existe, Gladys Memnon, Yves St. Victor, Bernard A. Okech, Connie J. Mulligan

**Affiliations:** 1 Genetics and Genomics Program, University of Florida, Gainesville, Florida, United States of America; 2 Department of Anthropology, University of Florida, Gainesville, Florida, United States of America; 3 Genetics Institute, University of Florida, Gainesville, Florida, United States of America; 4 Department of Medicinal Chemistry, University of Florida, Gainesville, Florida, United States of America; 5 National Public Health Laboratory, Port au Prince, Haiti; 6 Hospital Saint Croix, Leogane, Haiti; 7 Blanchard Clinic, Terre Noire, Haiti; 8 Emerging Pathogens Institute, University of Florida, Gainesville, Florida, United States of America; 9 Department of Environmental and Global Health, University of Florida, Gainesville, Florida, United States of America; London School of Hygiene and Tropical Medicine, UNITED KINGDOM

## Abstract

Hispaniola, comprising Haiti and the Dominican Republic, has been identified as a candidate for malaria elimination. However, incomplete surveillance data in Haiti hamper efforts to assess the impact of ongoing malaria control interventions. Characteristics of the genetic diversity of *Plasmodium falciparum* populations can be used to assess parasite transmission, which is information vital to evaluating malaria elimination efforts. Here we characterize the genetic diversity of *P*. *falciparum* samples collected from patients at seven sites in Haiti using 12 microsatellite markers previously employed in population genetic analyses of global *P*. *falciparum* populations. We measured multiplicity of infections, level of genetic diversity, degree of population geographic substructure, and linkage disequilibrium (defined as non-random association of alleles from different loci). For low transmission populations like Haiti, we expect to see few multiple infections, low levels of genetic diversity, high degree of population structure, and high linkage disequilibrium. In Haiti, we found low levels of multiple infections (12.9%), moderate to high levels of genetic diversity (mean number of alleles per locus = 4.9, heterozygosity = 0.61), low levels of population structure (highest pairwise F_st_ = 0.09 and no clustering in principal components analysis), and moderate linkage disequilibrium (ISA = 0.05, P<0.0001). In addition, population bottleneck analysis revealed no evidence for a reduction in the *P*. *falciparum* population size in Haiti. We conclude that the high level of genetic diversity and lack of evidence for a population bottleneck may suggest that Haiti’s *P*. *falciparum* population has been stable and discuss the implications of our results for understanding the impact of malaria control interventions. We also discuss the relevance of parasite population history and other host and vector factors when assessing transmission intensity from genetic diversity data.

## Introduction

Malaria remains a leading global health threat, accounting for 627,000 deaths in 2012. Reduced morbidity and mortality in the last decade as a result of increased financial support have renewed interest in pursuing global malaria eradication [1, 2]. Nineteen countries are now in malaria elimination or pre-elimination program phases while other the endemic countries remain in control phase as they assess the feasibility of elimination strategies. Hispaniola, home to Haiti and the Dominican Republic, is the only remaining Caribbean island with endemic malaria. The island has been identified as an ideal candidate for malaria elimination interventions [3–5] because Hispaniola: 1) has relatively low transmission of the malaria parasite [6–8], 2) has only one primary parasite species (*Plasmodium falciparum)* [9], 3) little to no antimalarial resistance exists as evidenced by drug efficacy studies and molecular studies [10–18] despite decades of chloroquine first-line treatment policy in Haiti, and 4) has a low risk of re-importation, a benefit of being an island surrounded by non-malaria endemic islands.

Given that the majority of malaria cases are reported in Haiti, elimination planning in Hispaniola focuses interventions in Haiti. Although Haiti is a strong candidate for malaria elimination, the 2014 World Malaria Report lists Haiti’s malaria program phase as “control” with the note “insufficiently consistent data to assess trends” [1]. Incomplete surveillance data are likely the result of Haiti’s limited public health infrastructure. In recent years, the public health system was further weakened by natural disasters, most notably the 2010 earthquake. Not only did the earthquake damage key government building and hospitals, it left many Haitians homeless and exposed to *P*. *falciparum*-carrying mosquitos. More cases of malaria infection were reported after the 2010 earthquake than before the earthquake, though it is unclear whether this increase was the result of increased exposure to *P*. *falciparum* or the result of increased surveillance by visiting aid groups in Haiti [6].

The genetic diversity of the *P*. *falciparum* population can be a useful tool to characterize parasite populations. The level of genetic diversity and the distribution of that diversity, or population structure, provide insight into trends in parasite transmission and parasite population history, information that is vital to malaria elimination programs seeking to assess the impact of elimination interventions. While advances in sequencing technology allow for whole genome-wide estimates of genetic diversity in *P*. *falciparum* populations [19], the more traditional microsatellite-based approach remains one of the most efficient methods for obtaining genetic diversity data on *P*. *falciparum* populations for epidemiological purposes. Microsatellites markers have been employed extensively to study *P*. *falciparum* population structure within countries and across continents [20]. In general, we see that parasites from human populations with low malaria transmission (<1% infection rate, as defined in Anderson et al.[20]) have less genetic diversity, more population structure and greater linkage disequilibrium (i.e. more non-random association among alleles across multiple loci), while parasites from high transmission populations (>1% infection rate) carry more genetic diversity, lower levels of population structure, and less linkage disequilibrium [20–23]. The reasoning behind these patterns is that individuals in high transmission populations are more likely to be infected by more than one *P*. *falciparum* parasite which increases the frequency of recombination and subsequently results in a highly diverse population with low linkage disequilibrium [19, 20]. In contrast, in populations where malaria transmission is low, increased inbreeding in parasite populations is expected to lead to less diversity and more parasite population structure. While these observations are mostly consistent across countries with different transmission intensities, some studies report deviations from this pattern. Specifically, high levels of heterozygosity, an estimate of genetic diversity, have been reported in several low transmission countries [24–26]. There is a suggestion that the high level of heterozygosity may reflect past human demographic processes as opposed to recent epidemiological factors [27]. More studies in diverse parasite–host ecological settings are needed to better understand the relationship between malaria transmission rates and *P*. *falciparum* genetic diversity and population structure.

Most *P*. *falciparum* microsatellite studies have focused on large geographic scales and it is unclear if the reported trends in population structure and genetic diversity are applicable on a local scale or in small, geographically isolated regions like Haiti. Our study examined microsatellite loci in the *P*. *falciparum* population in Haiti with the goal of understating the characteristics of genetic diversity of the malaria parasite population in Haiti. We discuss the results in light of what has been observed in other countries of either high or low transmission. Eighty-five dried blood spot samples collected between 2010 and 2013 from Terre Noire, Leogane, Jacmel, Chabin, Nippes, North Cap Haitian, and Hinche were assayed for 12 neutral microsatellite loci previously used to investigate *P*. *falciparum* genetic diversity [20].

## Methods

### Sample Collection and Study Sites Description

**Fig 1:**
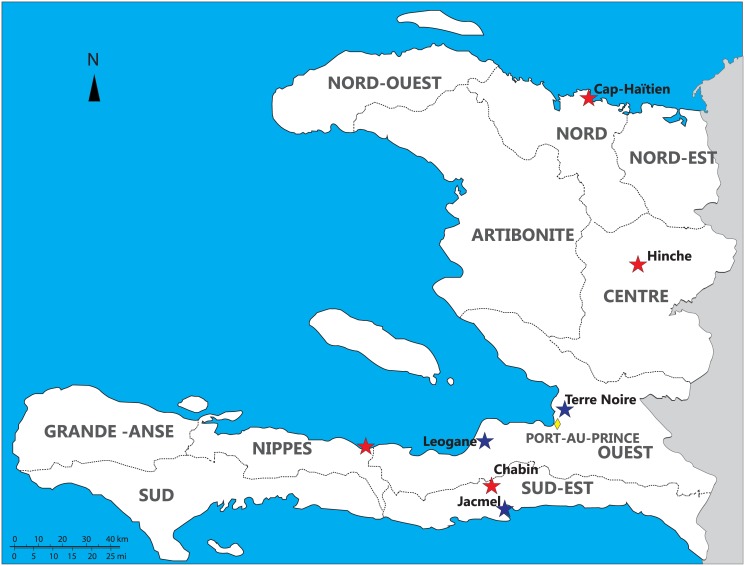
Map of study sites in Haiti. Study sites are indicated by the colored stars. Blue stars indicate the study sites where the majority of samples were collected.

Dried blood spot samples from patients suspected or confirmed to have malaria infections (by microscopy or rapid diagnostic test) were collected at health centers in seven locations throughout Haiti between May 2010 and March 2013 (Fig 1). The samples were collected in Terre Noire (within Port au Prince) (n = 39) and Leogane (n = 18) in the Ouest Department and in Jacmel (n = 18) in Sud Est department (Table 1). Other samples were collected from North Cap Haitien (n = 2), Hinche (n = 4), Nippes (n = 2), and from Chabin (n = 2). The communities are characterized by high rates of poverty. The distance between all sampling sites is less than 200 km. For the largest samples sets (Terre Noire, Leogane, and Jacmel), the distance is less than 50 km.

**Table 1:** Sample size across study sites and collection years.

Site	2010	2011	2012	2013	All
Terre Noire	6	21	12	0	39
Leogane	0	9	9	0	18
Jacmel	0	0	14	2	16
Chabin	0	0	0	2	2
Hinche	0	4	0	0	4
North Cap Haitien	0	4	0	0	4
Nippes	0	0	2	0	2
All Sites	6	38	37	4	85

Very little information is known about differences in transmission among the regions included in this study. While malaria transmission in Haiti is low overall, there have been reports of transmission hotspots in the Southeast Department (includes Jacmel and Chabin), particularly near coastal areas [28]. In this region, prevalence averages at 9.5%. A recent investigation into gametocyte carriage across departments in Haiti found the highest rates in Sud-est department [28]. Ouest (Terre Noire and Leogane) showed about 1.2% rate of gemtocyte carriage. Nippes Department (Nippes) reported 0.2% gametocyte carriage. No gametocyte data is available for the central and northern departments. In addition, no entomological based estimates of transmission variation within Haiti are available now due to limited infrastructure needed for continued surveillance, but investigations into this topic are now underway [5].

Sample collection between 2010 and 2012 has been previously described [10, 11]. Recent Jacmel and Chabin samples were collected in the same manner. For the present study, we examined only samples that were successfully genotyped for *dhfr*, *dhps*, *pfmdr*, or *pfcrt* genes indicating the presence of *P*. *falciparum*.

### Ethics statement

All malaria-infected patients were treated with chloroquine or another antimalarial drug as was the standard policy at the clinics. The study was approved by the Haiti Ethical Review Board, UF-IRB, and the Office of Research Protections, US Army Medical Research and Materiel Command (USAMRMC). Written consent was obtained by all participants as approved by the ethics committees listed above.

### DNA Extraction and Microsatellite Genotyping

DNA was extracted from dried blood spots using a Qiagen QIAmp Investigator Kit (Qiagen Inc., Chatsworth, CA). Between six to eight punches were taken for each blood spot and final DNA samples were eluted in 60μL elution buffer. Samples were assayed for 12 putatively neutral microsatellites loci previously used to characterize the genetic diversity *P*. *falciparum* populations worldwide: chromosome 4: Polya; chromosome 5: TA81 and TA42; chromosome 6: TA1, TA87, and TA109; chromosome 10: TA40 and 2490; chromosome 11: ARA2; chromosome 12: Pfg377 and PFPK2; and chromosome 13: TA60 [20, 29, 30]. A semi-nested PCR protocol and primer sequence have been previously described [30]. Our protocol was slightly modified, in that we used 25μL PCR reactions and did not multiplex the reactions. All PCR reactions included reagents at the following concentrations: 1X GoTaq Flexi Buffer, 2.5mM MgCl_2_, 0.2 mM each nucleotide, 0.25 μM for each primer in the primary PCR (0.4 μM in the semi-nested PCR), and 0.625 U of Go Taq Hot Start Polymerase (Promega, Madison, USA). The same primers were used as detailed in Anderson et al.[30], with the exception of TA40, which was replaced by the TA40 primers listed in a more recent protocol published by the University of Maryland CVD Malaria Group (CVD Malaria Group, University of Maryland, Sequences: TA40 Rev-1 GAAATTGGCACCACCACA, TA40 For AAGGGATTGCTGCAAGGT, TA40 Rev-2 CATCAATAAAATCACTACTA). Fragment analysis of PCR products were conducted at the University of Florida’s Interdisciplinary Center for Biotechnology Research DNA Genotyping Core Laboratory, using BigDye^TM^ chemistry on an Applied Biosystems 3730 Genetic Analyzer. Raw electropherograms were analysed using GeneMarker (Softgenetics, State College, USA). All peaks greater than 1,000 units were called.

### Data Analysis

#### Multiplicity of infection

Multiplicity of infection was defined as having more than one peak at one or more microsatellite loci (*P*. *falciparum* genome is haploid while in human hosts) Minor peaks were not counted if they were less than 1/3 the height of the major peak. We determined the number of multiple infections in the overall sample set, by study sites, and by collection year. For genetic diversity analyses, only the major allele at each locus in multiple infected samples was included.

#### Genetic diversity

Genetic diversity was evaluated by the number of alleles at each locus and heterozygosity (H_e_). We determined H_e_ and number of alleles for each locus and the mean values by study site (Terre Noire, Leogane, and Jacmel only) and by year of collection (2011 and 2012 only). Other study sites and collections years were not included due to small sample sizes. We also determined the number of haplotypes, defined as having 12 identical microsatellite alleles. To do this, we only included samples with no more than two alleles per locus and only the major alleles/tallest peaks were used for haplotype determination.

#### Effective population size and population bottleneck analysis

To evaluate differences in population size at different locations and across time, we estimated effective population size by site and by year of collection based on an infinite-alleles model (IAM) and a stepwise mutation model (SMM), since both patterns of variation have been reported for our microsatellite assay [20, 31]. Estimates were calculated as reported in Anderson et al., [31, 32]. We also tested for evidence of a population bottleneck using a simulation-based approach in BOTTLENECK1.2.02 [33].

#### Linkage disequilibrium

To assess whether alleles from different loci were associated with each other, we tested for linkage disequilibrium (LD) using LIAN [34]. A measure of LD, the standardized index of association (I^S^_A_), was calculated and can be used for across-study comparisons. For our study, we completed 10,000 resamplings.

#### Population substructure

To investigate population substructure, we determined the number of private alleles, i.e. alleles found in only one study site or collection year, and then examined geographic substructure by calculating pairwise F_st_ between Terre Noire, Leogane, and Jacmel. Pairwise F_st_ and accompanying p-values were calculated in Arlequin v3.5x [35]. We also preformed principal component analysis (PCA) to detect non-geographically constrained sub-populations in our samples using SAS^®^ 9.2 software (SAS Institute Inc, Cary, USA).

## Results

### Sample Size

Ninety-four samples were selected for genotyping based on previous successful genotyping of *P*. *falciparum dhfr*, *dhps*, *pfmdr*, or *pfcrt* genes. In total, 85 samples were successfully genotyped for least 10 of the 12 microsatellite loci and were included in the final sample for analysis (S1 Table). Of the 85 samples, 76 samples were genotyped for all 12 microsatellite loci. The majority of samples (73/85) were collected from Terre Noire, Leogane, and Jacmel (Table 1) and 75/85 samples were collected within a ten month period between September 2011 and June 2012.

### Multiplicity of Infection

Table 2 lists multiple infections, mean number of alleles (ie. mean number of alleles across all 12 loci), and heterozygosity in Haiti, as well comparative data previously reported in populations with low (Colombia, Brazil, Bolivia, and Thailand) and high (Zimbabwe, Uganda, Congo, and Papua New Guinea) malaria transmission [20], Of the 85 samples collected, 11 multiple infections (12.94%) were identified. Five of the 11 multiple infections had more than one locus with multiple alleles (data not shown). The percentage of multiple infections within our three largest sites were as follows: 10.26% (0.73–19,78 95% CI) in Terre Noire, 16.67% (-0.55, 33.88% 95% CI) in Leogane, and 25.00% (3.78, 46.22 95% CI) in Jacmel ranged from 10 to 25% (multiple infections were only observed in Terre Noire, Leogane, and Jacmel). We also report the multiple infections by collection year (S2 Table), though sample sizes were too small to make statistical comparisons.

**Table 2:** Multiplicity of infection, mean number of alleles, and heterozygosity in Haiti compared to ranges observed in high and low malaria transmission populations.

	n	No. multiple infections–at least one locus with multiple alleles	Percent Multiple Infection(95% Confidence Intervals)	Mean No. of alleles(Standard deviation)	Heterozygosity (Standard deviation)
Haiti	85	11	12.94 (5.81,20.08)	4.92 (± 1.68)	0.61 (± 0.13)
**Other countries by transmission intensity** ¥					
Low Transmission	—	—	<20.00%	2.17–4.92	0.30–0.40
High Transmission	—	—	>45.00%	6.00–10.67	0.62–0.80

^¥^ Data reported from Anderson et al.[20].

### Genetic Diversity

**Fig 2:**
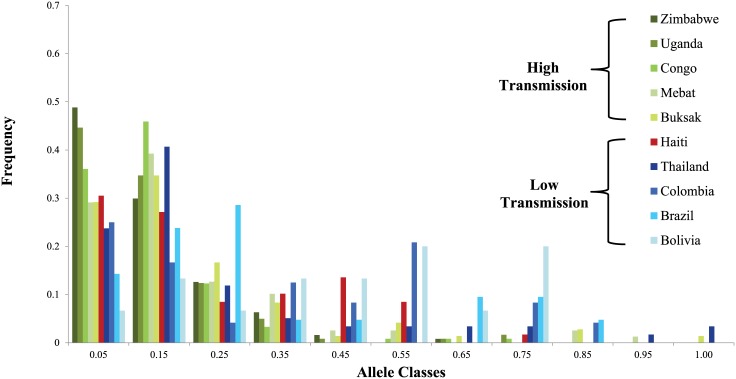
Allele frequency distribution in Haiti compared to high and low malaria transmission countries. Non-Haitian data taken from Anderson et al.[20]. Mebat and Buksak are sites within Papua New Guinea.

The mean number of alleles observed was 4.92 (± 1.68), with a range of 2 (2490) to 7 (TA81) alleles (see S3 Table for mean number of alleles for each microsatellite). The mean H_e_ was 0.61 (standard deviation = 0.13), with a range of 0.28 (TA42) to 0.73 (Polyα and PFPK2). The mean heterozygosity (standard deviation in parenthesis) within study sites was 0.57 (0.17) in Terre Noire, 0.59 (0.14), in Leogane, and 0.57 (0.19) in Jacmel. Table 2 lists the mean number of alleles and heterozygosity (H_e_) for Haiti overall and for high and low transmission countries previously investigated [20]. Mean number of alleles and heterozygosity are reported across collection years (2011 and 2012 only) in S2 Table. We also plotted a comparison of the distribution of allele frequencies observed in Haiti’s *P*. *falciparum* population to populations from low and high transmission countries reported in Anderson et al.[20] (Fig 2). In high transmission populations, the distribution takes on more of an L shape, with more low frequency alleles observed when compared to low transmission populations, which have more high frequency alleles. The distribution of allele frequencies in Haiti did not resemble either low or high transmission populations, rather following a more intermediate pattern.

We determined the number of private alleles (i.e. an allele detected at only one study site). Four study sites carried private alleles at five loci; of those five loci, four contained only a single unique allele (data not shown). TA60 had two private alleles only observed in Terre Noire. Most private alleles were detected only in a single individual with the exception of the TA40 199 allele, which was observed in four individuals from Jacmel.

We examined the number of microsatellite haplotypes in our study, excluding samples with any missing genotypes or greater than two alleles at any locus. Of 76 eligible samples, 62 haplotypes were observed (data not shown). Seven haplotypes were observed in more than one sample, while the remaining 55 haplotypes were only observed once. One haplotype was detected in six samples across two study sites (H1; Terre Noire = 5, Leogane = 1) and across three collections years (2010 = 2, 2011 = 1, and 2012 = 3). The other multiple sample haplotypes were found in 2–3 samples each, which limited the number of comparisons that could be done across study sites or collection year.

### Effective Population Size and Bottleneck Analysis

Overall, we calculated an effective population size of 2084 to 3891 individuals across all samples, based on the infinite alleles (IAM) and single stepwise mutation (SMM) models, respectively. Effective population size by study site and year are reported in S4 Table. We tested for evidence of a population bottleneck by comparing the heterozygosity calculated from the number of alleles to the observed H_e_ reported above. Although both H_e_ and number of alleles decline after a population bottleneck, H_e_ generally declines at a slower rate so excess H_e_ is suggestive of a recent population bottleneck. In our study, we found no significant deviation of H_e_ based on the observed number of alleles under the stepwise mutation model (Wilcoxon test for heterozygosity excess, p-value = 0.883), suggesting no recent history of population bottlenecks.

### Linkage Disequilibrium

We examined evidence of linkage disequilibrium (LD) between microsatellite markers for the *P*. *falciparum* samples from Haiti using a Monte Carlo simulation method to calculate the standard index of association. We found significant levels of LD in the total data set (I^S^_A_ = 0.051, p = < 0.0001). LD was also detected within the three largest study sites: I^S^_A_ = 0.086 for Terre Noire (p <0.0001), I^S^_A_ = 0.095 for Leogane (p <0.0001), and I^S^_A_ = 0.041 for Jacmel (p <0.001).

### Population Substructure

**Fig 3:**
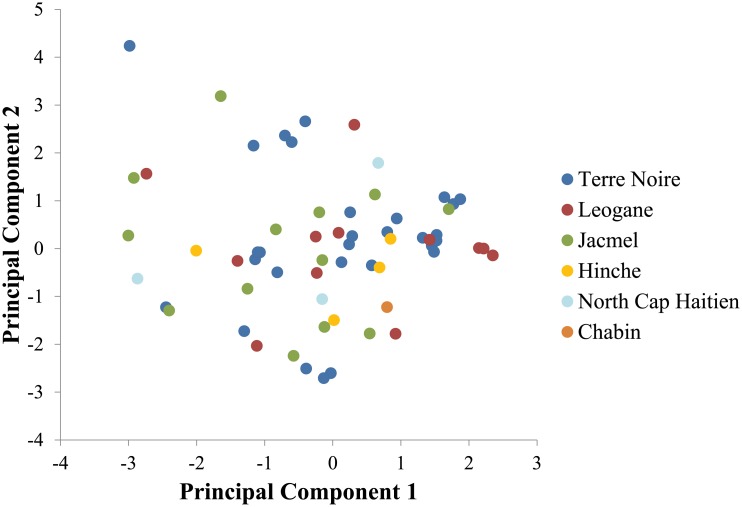
Plot of the first two principal components of principal component analysis, color coded by study site.

We tested for geographic population substructure by calculating pairwise F_st_ across the three largest study sites (Table 3). Overall, virtually no population substructure was observed between study sites as determined by low F_st_ values; F_st_ values ranged from 0.02 to 0.09. We also used principal component analysis to examine our data for evidence of population substructure. Principal components were plotted and no clustering, geographic or otherwise, was observed in our samples (Fig 3).

**Table 3:** Analysis of population structure across three largest sample sites: Terre Noire (Port au Prince), Leogane, and Jacmel. Pairwise F_st_ (bottom half) and p-values (top half) are listed.

Study Sites	Terre Noire	Leogane	Jacmel
Terre Noire	—	0.04505	<0.00001
Leogane	0.02488	—	0.00901
Jacmel	0.09288	0.06959	—

## Discussion

Our study employed the use of a commonly used multi-locus microsatellite assay to characterize *P*. *falciparum* genetic diversity in Haiti. Previous studies have shown that low levels of multiple infections and genetic diversity (H_e_ and number of alleles), combined with high levels of linkage disequilibrium and population structure (estimated with F_st_ and PCA), are evidence of low transmission populations, while the reverse is observed in high transmission populations. We found that the frequency of multiple infections and presence of linkage disequilibrium (LD) in our study resembled countries with low malaria transmission, but other diversity-related estimates, such as heterozygosity and lack of population structure, resembled higher transmission countries. Specifically, the small proportion of multiple infections in our sample (12%) is similar to the prevalence of multiple infections in other low transmission countries (2–25% in Columba, Bolivia, and Brazil [20]) and confirm findings in a previous investigation of multiple infections in Haiti using the merozoite surface protein 1 and 2 genes [36]. The mean number of alleles across microsatellite loci (4.9) was on the higher end of the range observed in most studies of low transmission countries (2.42–4.92 alleles in Columbia, Bolivia, Brazil, Honduras, Peru, and Thailand [20, 23, 25]), but lower than high transmission countries (6–14 alleles in Uganda, Congo, Zimbabwe, Papua Guinea [20, 37]). We also found a significant level of LD across microsatellite loci in Haiti’s *P*. *falciparum* population (I^S^_A_ = 0.05, p-value < 0.0001), falling at the lower end of the range observed in other low transmission populations (0.02–0.14 in Columba, Bolivia, Brazil, Thailand, and Hondorous [20, 23]). The LD observed could be due to household clustering resulting from uninterrupted feeding, though additional studies are needed to confirm that this would may a significant contribution at the population level. Virtually no geographic substructure was detected in Haiti based on F_st_ (F_st_ ranged from 0.02 to 0.09). It is worth noting that pairwise F_st_ values can vary within a region of low transmission, as seen in Brazil, with pairwise Fst values ranging from 0.04 to 0.30. Given that the greatest physical distance between sites was 100km, with our three largest sites being within 40km of each other, high levels of substructure would not be expected [38]. Finally, our principal component analysis (Fig 3) support the results of the Fst values, indicting no geographic substructure within Haiti suggesting free gene flow across study sites. In sum, Haiti’s *P*. *falciparum* genetic diversity characteristics do not fall cleanly into what would be expected for a high or low transmission population.

An unexpected result of this study was the high level of heterozygosity observed, suggesting a larger *P*. *falciparum* population than expected for a low transmission country. The level of H_e_ across loci (0.61) is suggestive of high malaria transmission countries, as evidenced by previous studies in high transmission populations (H_e_ = 0.62–0.80 in Papua New Guinea, Uganda, Congo, Zimbabwe, [3]). Several other studies have reported higher levels of heterozygosity than expected for a low transmission country [25, 26, 39], questioning whether heterozygosity directly correlates with transmission. The high level of heterozygosity does suggest a large effective parasite population size. This finding along with the fact that we did not detect a reduction in population size, based on population bottleneck analysis looking at variance in allele frequencies, could suggest that Haiti’s *P*. *falciparum* population has been relatively stable over time. It is worth noting no decline in effective population size has been observed in populations that have undergone increased interventions [40] which may be a result of a limited ability to detect changes in a large effective population size. Additional studies into *P*. *falciparum* populations compared to our findings can confirm whether parasite population size is changing.

Given the lack of consistent resources for controlling malaria in Haiti, our results may support the notion that the *P*. *falciparum* population has not been impacted by interventions such as mosquito bed nets, insecticides, and antimalarial drug use. As mentioned above, widespread antimalarial resistance is not reported in Haiti, further supporting the idea that interventions like antimalarial drugs have not been used to the level that would have a significant impact on the overall *P*. *falciparum* population size in Haiti. These results highlight the importance of considering the extent to which long-term regional malaria control interventions have, or have not, impacted parasite evolution and population size when making inferences about transmission intensity from *P*. *falciparum* population genetic diversity data.

In addition, we must consider the role that host genetic diversity has on the parasite population, specifically the role that inherited protective red blood cell disorders, such as sickle cell hemoglobin and glucose-6-phosphate dehydrogenase deficiency, may play in shaping each parasite population. It is worth noting that the initial correlation between genetic diversity measures and transmission intensity across continents was also a comparison across host populations with different genetic backgrounds [20]. In Haiti, a host population with high West African ancestry, we observe a high frequency of malaria protective red blood cell polymorphisms [41, 42], which may play a role in shaping the parasite genetic diversity. It is interesting that we see lower frequencies of these protective polymorphisms in lower transmission regions in South America, which may explain part of the inconsistency between our results and other lower transmission populations. In addition to host related factors, characteristics of the vector could maintain *P*. *falciparum* diversity. If multiple vector species exist, multiple parasite strains could evolve to adapt to each vector species. Only one malaria vector species has been confirmed in Haiti, but it possible that more parasite vector species exist. Future studies should determine if additional vector species exist in Haiti and determine what impact, if any, this has on the diversity of the parasite populations in Haiti.

Sample size was a limitation of our study. Most of our sample was collected from the southern portion of Haiti, which may limit our ability to extrapolate our results to the rest of the country. Our across-site comparisons with principal component analysis, however, showed that the smaller sample sets from Hinche, Cap Haitien, Nippes, and Chabin clustered with the larger sample sets, suggesting that geographic population substructure in Haiti may be minimal and *P*. *falciparum* gene flow may be relatively unimpeded. Further studies with larger samples collected across multiple sites in Haiti are needed to confirm our observations. Our study in Haiti highlights some of the challenges that population genetic and epidemiological studies face when working with small endemic populations, particularly those with low malaria transmission and very few number of cases detected and reported.

In conclusion, we found that the characteristics of Haiti’s *P*. *falciparum* population do not fall neatly within expectations for either a low or high transmission population. While the proportion of multiple of infections is suggestive of a low transmission population, we see high allelic diversity similar to that seen in high transmission regions. Our investigation into population bottlenecks in Haiti showed no decline in effective population size, suggestive that the parasite population may be relatively stable. It is possible that other potential for factors other than transmission intensity may shape the diversity of the parasite population, such as the history and extent of malaria control interventions and the high frequency of protective red blood cell disorders in the host populations. The results of this study injunction with continued surveillance of the genetic diversity of *P*. *falciparum* population in Haiti may provide further insight as to whether recently adopted malaria control policies are successfully reducing parasite population in Haiti.

## Supporting Information

S1 Table Microsatellite genotypes and allele codes. (XLSX)

S2 Table Multiplicity of infection, mean number of alleles, and heterozygosity by collection year. (DOCX)

S3 Table Number of alleles and heterozygosity across microsatellite loci. (DOCX)

S4 Table Effective population size, N_e,_ across study site and collection year. Estimates were calculated based on both the infinite-allele (IAM) and the stepwise (SMM) mutation models. (DOCX)
