# Supplementary material for: Genetic Diversity of Plasmodium falciparum in Haiti: Insights from Microsatellite Markers
Source: PLoS One. 2015 Oct 13;10(10):e0140416. doi: 10.1371/journal.pone.0140416 (PMC4604141; doi:10.1371/journal.pone.0140416)
Supplement: S2 Table — (DOCX) [file pone.0140416.s002.docx]

**S2 Table. Multiplicity of infection, mean number of alleles, and heterozygosity by collection year.**

|  | **n** | **No. multiple infections – at least one locus with multiple alleles** | **Percent Multiple Infection**  **(95% Confidence Intervals)** | **Mean No. of alleles**  **(Standard deviation)** | **Heterozygosity (Standard deviation)** |
| --- | --- | --- | --- | --- | --- |
| **By Collection Year** |  |  |  |  |  |
| 2010 | 6 | 1 | 16.67 (-13.15, 46.49) | -- | -- |
| 2011 | 38 | 3 | 7.89 (-0.68, 16.46) | 4.42 (±1.38) | 0.61(±0.13) |
| 2012 | 37 | 6 | 16.22 (4.34, 28.1) | 4.25 (±1.14) | 0.62 (±0.12) |
| 2013 | 4 | 1 | 25 (-17.43, 67.43) | -- | -- |
