# Supplementary material for: Genetic Diversity of Plasmodium falciparum in Haiti: Insights from Microsatellite Markers
Source: PLoS One. 2015 Oct 13;10(10):e0140416. doi: 10.1371/journal.pone.0140416 (PMC4604141; doi:10.1371/journal.pone.0140416)
Supplement: S3 Table — (DOCX) [file pone.0140416.s003.docx]

**S3 Table. Number of alleles and heterozygosity across microsatellite loci.**

| **Locus** | **Sample size** | **No. of Alleles** | **H_e_** |
| --- | --- | --- | --- |
| **TA1** | 83 | 4 | 0.68 |
| **TA60** | 83 | 6 | 0.61 |
| **Polya** | 83 | 8 | 0.73 |
| **ARA2** | 84 | 5 | 0.62 |
| **Pfg377** | 85 | 4 | 0.62 |
| **TA81** | 84 | 7 | 0.56 |
| **TA42** | 83 | 3 | 0.28 |
| **PFK2** | 85 | 6 | 0.73 |
| **_2490** | 85 | 2 | 0.50 |
| **TA109** | 84 | 4 | 0.59 |
| **TA87** | 85 | 5 | 0.64 |
| **TA40** | 84 | 5 | 0.72 |
| **Mean** | 84 | 4.92 | 0.61 |
| **Standard deviation** | 0.85 | 1.68 | 0.13 |
