# Supplementary material for: Genetic Diversity of Plasmodium falciparum in Haiti: Insights from Microsatellite Markers
Source: PLoS One. 2015 Oct 13;10(10):e0140416. doi: 10.1371/journal.pone.0140416 (PMC4604141; doi:10.1371/journal.pone.0140416)
Supplement: S4 Table — (DOCX) [file pone.0140416.s004.docx]

**S4 Table. Effective population size, N_e,_ across study site and collection year. Estimates were calculated based on both the infinite-allele (IAM) and the stepwise (SMM) mutation models.**

|  | **IAM - N_e_** | **Confidence Intervals** | **SMM - N_e_** | **Confidence Intervals** |
| --- | --- | --- | --- | --- |
| **All** | 2084 | (1057, 5602) | 3891 | (1883, 9983) |
| **Study Site** |  |  |  |  |
| Terre Noire | 2084 | (896, 4748) | 3466 | (1489, 7895) |
| Leogane | 2263 | (972, 5154) | 3891 | (1672, 8863) |
| Jacmel | 2084 | (896, 4748) | 3466 | (1489, 7895) |
| **Study Year** |  |  |  |  |
| 2011 | 2459 | (1057, 5602) | 4383 | (1883, 9983) |
| 2012 | 2565 | (1102, 5844) | 4658 | (2002, 10611) |
